# Evaluation of the impact of telementoring using ECHO© technology on healthcare professionals’ knowledge and self-efficacy in assessing and managing pain for people with advanced dementia nearing the end of life

**DOI:** 10.1186/s12913-018-3032-y

**Published:** 2018-04-02

**Authors:** Bannin De Witt Jansen, Kevin Brazil, Peter Passmore, Hilary Buchanan, Doreen Maxwell, Sonja J. McIlfatrick, Sharon M. Morgan, Max Watson, Carole Parsons

**Affiliations:** 1 School of Pharmacy, Queen’s University Belfast, 97 Lisburn Road, Belfast, BT9 7BL UK; 2 School of Nursing and Midwifery, Queen’s University Belfast, Belfast, UK; 3 Centre for Public Health, School of Medicine, Dentistry and Biomedical Sciences, Queen’s University Belfast, Belfast, UK; 4 Patient and Public Involvement Representative, Carer for a person living with dementia, Belfast, UK; 5 Kerrsland Surgery, Belfast, UK; 6 Institute of Nursing and Health Research, Ulster University, Coleraine, UK; 7 All Ireland Institute of Hospice and Palliative Care, Our Lady’s Hospice and Care Services, Dublin, Ireland; 8 Marie Curie Hospice, Belfast, UK; 9 Northern Ireland Hospice, Belfast, UK

**Keywords:** Dementia, Palliative care, Pain assessment, Pain management, Telementoring, ECHO©, Knowledge, Self-efficacy

## Abstract

**Background:**

Pain assessment and management in advanced and end-stage dementia are challenging; patients are at risk of under-diagnosis, under-assessment and under-treatment. Previous research has highlighted the importance of needs-driven training and development in this area for physicians, nurses and healthcare assistants (HCAs) across specialties, disciplines and care settings. This study used teleconferencing technology to connect healthcare professionals across multiple settings and disciplines in real-time clinics, based on the Project ECHO© model. This paper reports the evaluation of the clinics by physicians, nurses and HCAs, including their knowledge and self-efficacy in pain assessment and management for patients with advanced and end-stage dementia.

**Methods:**

A mixed method evaluation comprising quantitative survey of self-reported knowledge and self-efficacy pre- and post-ECHO clinic participation, and qualitative exploration of experiences of the clinics using focus group interviews. A census approach to sampling was undertaken. Pre- and post-ECHO evaluations were administered electronically using Survey Monkey software. Mann-Whitney U tests were used to explore differences in knowledge and self-efficacy scores pre- and post-ECHO clinic participation. Statistical significance was set a-priori at *p* = 0.05. Focus groups were video- and audio-recorded, transcribed verbatim and analysed using Braun & Clarke’s model of thematic analysis.

**Results:**

Eighteen healthcare professionals [HCPs] (physicians [*n* = 7], nurses [*n* = 10], HCA [*n* = 1]) and twenty HCPs (physicians [*n* = 10], nurses [*n* = 10]) completed pre- and post-ECHO evaluations respectively, reporting improvements in knowledge and self-efficacy on participation in ECHO clinics and perceived utility of the clinics. Seven HCPs (physicians [*n* = 2], nurses [*n* = 5]) participated in two focus groups. Four themes emerged: knowledge and skills development and dissemination; protected time; areas for improvement; and the future of ECHO.

**Conclusions:**

Telementoring clinics for HCP education and training in pain assessment and management in advanced and end-stage dementia demonstrate a positive impact on knowledge and self-efficacy of HCPs and highlight the value of a cross-specialty network of practice which spans across disciplines/HCP types, care settings and geographical areas. Further development of ECHO services in this and in other clinical areas, shows significant potential to support delivery of high-quality care to complex patient populations.

**Electronic supplementary material:**

The online version of this article (10.1186/s12913-018-3032-y) contains supplementary material, which is available to authorized users.

## Background

The advanced stages of dementia are characterised by immobility, severe cognitive deficit, loss of communication skills, and physical frailty, and are often accompanied by distressing and/or painful symptoms including: respiratory infection, delirium, anorexia, dysphagia, incontinence and sleep disturbance [1–4]. Research evidence suggests that people who are dying with dementia are liable to experience pain at the end of life [5, 6]; studies indicate that between 20 and 50% of people with dementia report some form of pain in the course of their illness progression [7], with higher proportions affected in the more advanced stages of the condition and towards the end of life [8–13]. Pain recognition and assessment in this patient population is widely recognised to be challenging; extensive cognitive decline in the advanced and terminal stages of dementia often significantly impair or remove the possibility of patient self-report, increasing the risk of under-assessment and under-treatment of pain [14–18].

It was in this context that a programme of research into assessing and managing pain in people with advanced dementia nearing the end of life was undertaken to determine the issues in assessment and management of pain in this patient population, considering the perspectives of healthcare professionals (HCPs: physicians, nurses and healthcare assistants [HCAs] practising in primary, secondary and hospice care) and carers in order to develop a model of practice to optimise detection and treatment of pain as patients with dementia approach the end of life. The findings from the qualitative interview phase of this research programme have been presented in a number of peer-reviewed articles [19–21], and indicated the need for training and ongoing professional development for these HCPs (physicians, nurses and healthcare assistants) across specialties, disciplines and care settings. All respondents expressed a strong preference for case-based learning led by a health professional with clinical experience of the patient population. Learning by experience, sharing disciplinary knowledge, and opportunities to co-manage complex patient cases were seen to be key elements of a highly dynamic and relevant form of clinical training capable of cultivating sustained practice change.

Originally launched in 2003, Project ECHO© (Extension for Community Healthcare Outcomes) is a distance health education model which uses teleconferencing technology to connect HCPs across multiple setting and disciplines in real time clinics [22–25]. It uses a “hub and spoke” model, in which the ‘hub’ is the central physical location from which a specialist team hosts the clinic and the ‘spokes’ are HCPs who dial in remotely from their workplace. These HCPs typically include physicians, nurses and other health and allied health professionals working in areas relevant to the topic of the clinic. Specialist clinicians with relevant patient experience and clinical knowledge provide brief, focused didactic training on the clinical area, after which spoke members (typically one or two) present anonymised real patient cases for discussion. These discussions provide an opportunity for shared decision-making between the specialists at the hub and the spoke members. Project ECHO© has been trialled and evaluated across a range of health conditions and has demonstrated continued success in increasing substantive knowledge and professional self-efficacy, improving patient outcomes, and promotion of primary and secondary care integration [22–33]. To date, no evaluation of the Project ECHO© Model for pain assessment and management in advanced and end-stages of dementia has been undertaken.

This study therefore aimed to evaluate the impact of delivering education and training using the Project ECHO© Model on physicians’, nurses’ and HCAs’ self-reported clinical knowledge and self-efficacy in pain assessment and management in advanced and end-stages of dementia.

## Objectives

The objectives of the evaluation were:

- ➢ to analyse physicians’, nurses’ and HCAs’ scores from self-reported evaluations of clinical knowledge and self-efficacy in pain assessment and management in advanced and end-stages of dementia;
- ➢ to explore participants’ experiences of teleECHO professional mentoring, its perceived impact on practice change and utility of the ECHO pain clinic in pain management across health conditions and patient populations.

## Methods

Five TEAM Pain AD [Telementoring to Enhance Assessment and Management of Pain in Advanced Dementia] teleECHO clinics were held in June and July 2016 in the Project ECHO© Northern Ireland (Project ECHO© NI) superhub in Northern Ireland. The curriculum, (including the number of sessions and the topics covered), dates, lengths and times of the clinics were determined by key stakeholders and potential participants at a pre-ECHO workshop held in April 2016. Individuals invited to participate in this workshop included physicians, nurses and HCAs who had participated in the previous qualitative interview phase of this research which examined their experiences and perspectives of pain assessment and management in advanced dementia [19–21]. Other health and social care teams in primary, secondary, nursing home and hospice care settings and other key stakeholders were also invited to attend, and all who attended were invited to register their interest in participating in the TEAM Pain AD teleECHO clinics. All individuals who expressed an interest in participation were recruited to take part in the clinics, and individuals participated in as many or as few teleECHO sessions as they desired.

Each clinic was facilitated by the principal investigator (CP) at the hub, with participants attending at the hub or at “spokes” in their place of work using video-conferencing technology (Zoom Web Conferencing software, Zoom Video Communications, Inc., USA). Each session included a 20-min didactic training session on the specific topic area (Table 1) and participants were given an opportunity to ask questions. Patient case presentations then followed. These cases were distributed prior to each session using a standardised proforma, with patient confidentiality ensured. Cases were presented by a physician/nurse responsible for the care and/or management of the patient. The facilitator then opened case discussion to all clinic participants, which continued until a proposed treatment plan was outlined and/or sufficient guidance to address the clinical questions posed was provided. At the close of discussion, the facilitator summarised the proposed treatment plan/guidance. Each clinic lasted 1 h 15 min and was digitally recorded using video with audio.

**Table 1:** Curriculum for TEAM Pain AD teleECHO clinics

ECHO clinic	Topic
1	Managing challenges of routes of administration in pain management for people with advanced dementia (inc. managing non-compliance)
2	Non-pharmacological aspects of pain management in advanced dementia (inc. working with families, managing BPSD and distress)
3	Pain assessment in advanced dementia (inc. diagnosing pain, integrating pain assessment tools into clinical practice, clinical utility, limitations and practicality of assessment tools)
4	Pharmacology in advanced dementia (inc. polypharmacy, drugs to avoid, identifying and managing side and adverse effects)
5	Differentiating the behavioural indicators of pain from anxiety, agitation and other non-pain related behaviours in dementia

*BPSD* Behavioural and psychological symptoms of dementia

### Study population and sample

A census approach to sampling was undertaken; all physicians, nurses and HCAs in primary, secondary, nursing home and hospice care settings who participated in the TEAM Pain AD teleECHO clinics were approached to complete the evaluation forms and focus group discussion. Other health and allied health professionals such as speech and language therapists, occupational therapists, and pharmacists attended clinics but were not participants of the evaluation. Participants’ anonymity was assured. All who agreed to participate were included in the final sample. Focus groups were conducted until data saturation occurred.

### Study design

A mixed methods evaluation of teleECHO clinics in assessment and management of pain in patients with advanced dementia nearing the end of life, using a combination of quantitative questionnaires and qualitative focus group interviews as follows:

### Baseline assessment

Prior to the first TEAM Pain AD teleECHO clinic, physicians, nurses and HCAs registered for participation in the clinics were sent an email containing a link to complete a pre-ECHO online evaluation using Survey Monkey software (https://www.surveymonkey.co.uk). This collected data on demographic characteristics and self-reported evaluation of clinical knowledge and self-efficacy in pain assessment and management in advanced dementia nearing end of life. Submission of completed evaluation forms was deemed to constitute consent to participate in the evaluation.

### Post-ECHO assessment

Each participant was asked to complete an assessment of clinical knowledge and self-efficacy following the final ECHO clinic. This evaluation also contained items relating to participants’ experiences and perceptions of the utility of the teleECHO model. As at baseline, this was administered electronically using Survey Monkey software, and submission of completed evaluation forms was deemed to constitute consent to participate.

### Focus group

Two focus groups were held upon completion of the final ECHO clinic, to explore physicians’, nurses’ and HCAs’ experiences of the clinics. A topic guide (Table 2) was used to guide discussion and covered: reasons for participation in the TEAM Pain AD teleECHO clinics; perceptions of the efficacy of the curriculum (cases and didactic materials) in addressing learning needs; application of learning gained through TEAM Pain AD teleECHO clinics to patient care; impact of participation in the TEAM Pain AD teleECHO clinics on participants’ clinical teams; how, when, and if participants shared knowledge and skills from TEAM Pain AD teleECHO clinics with others; and participants’ perceptions of future ECHO pain clinics (e.g. the sustainability and utility of a central ECHO pain clinic that would cover pain across all heath conditions and patient populations).

**Table 2:** Topic guide for focus group interviews

1. Tell us about your experiences of participating in the TEAM Pain AD teleECHOs.
2. What were your reasons for participating in the TEAM Pain AD teleECHO clinics?
3. What did you like about the TEAM Pain AD clinics? What did you not like?
4. Did the curriculum (including the cases and didactic materials) address your learning needs? If so, in what way? If not, why not?
5. Do you think the teleECHO model can address the learning needs of healthcare professionals?
6. What are your thoughts on the range of didactic trainers and patient cases provided?
7. What are your thoughts on the varied audience of TEAM Pain AD clinics? Do you see a need or benefit to holding discipline-specific clinics?
8. Did you gain any clinical knowledge or skills through participation in TEAM Pain AD teleECHO clinics?
9. Have you applied any of the learning gained through TEAM Pain AD to your patients? If so, in what way? If not, why?
10. Have you shared any knowledge gained through TEAM Pain AD with other colleagues and care staff? If so, how did you do this? Has it made any difference to pain assessment and management in your care setting? In what ways?
11. What was the impact of your participation in TEAM Pain AD on your clinical teams in terms of staffing, workload and capacity? Is there anything we would need to consider when planning future ECHOs?
12. What are your thoughts on the future of teleECHO clinics: do you see a need for continuing pain clinics in dementia? How about for other chronic conditions?
13. Is there anything that would prevent you from participating in future teleECHO clinics?
14. Do you have any additional comments and/or feedback?
15. Is there anything you would like to ask us about the teleECHO clinics and/or the study?

Focus group discussions were video-recorded and audio data transcribed verbatim, checked and verified for accuracy. Written informed consent was sought prior to participation in the focus group interviews.

## Setting

### Data collection

Three versions of the pre-and post-ECHO questionnaires were designed to reflect the knowledge and self-efficacy domains pertinent to physicians, nurses and HCAs. These were developed using adapted material from the KnowPain-50 and KnowPain-12 questionnaires [34, 35], evaluations used by the original developers of Project ECHO© [22], items from the Palliative Care Evaluation Tool Kit [36], and following discussion and agreement by the Project Management Team (PMG; a group comprising two practising academic-physicians in geriatrics/dementia and palliative care, four academics specialising in palliative care, nursing and pharmacy, three General Practitioners (GPs) with a special interest in older adults, dementia and palliative care, and one patient and public involvement representative). Post-ECHO questionnaires also gathered data on participants’ experiences and perceptions of the utility of the teleECHO model.

Pre- and post teleECHO knowledge and self-efficacy scores were calculated for each respondent by summing scores for each statement, using 1 = Strongly Disagree; 2 = Disagree; 3 = Neither Agree nor Disagree; 4 = Agree; and 5 = Strongly Agree. Possible scores for this measure ranged from 14 to 70 for physicians, 11 to 55 for nurses, and 7 to 35 for HCAs. Measures for physicians, nurses and HCAs differed in the number and content of statements to reflect the remit of the target population. The physician questionnaire contained 14 items examining confidence in recognising and assessing pain, diagnosis, differentiating pain from behavioural and psychological symptoms of dementia (BPSD), prescribing via a range of routes of administration, assessing treatment response, clinical knowledge and self-efficacy, and using best practice approaches to assessing and managing pain. The nurse questionnaire contained 11 items which considered recognising and assessing pain, reporting pain, differentiating pain from BPSD, administering analgesia via a range of routes of administration, assessing treatment response, suggesting alternative formulations when the oral route is not available, recognising and managing breakthrough pain, discussing unresolved pain, clinical knowledge and self-efficacy and using best practice approaches to assessing and managing pain. The HCA questionnaire comprised 7 items considering recognising and reporting pain, differentiating pain from challenging behaviour, and discussing pain assessment and management with physicians and nurses. These items are detailed in full in Additional file 1: Table S1, Additional file 2: Table S2 and Additional file 3: Table S3.

### Data analysis

Descriptive statistics were used to describe and summarise participant characteristics. Mann Whitney U tests were used to explore differences in pre- and post-teleECHO evaluations and *p*-values reported to provide an indication of the impact of the model on HCPs’ self-reported clinical knowledge and self-efficacy. Statistical significance was set a-priori at *p* = 0.05.

Focus group interviews were transcribed verbatim, transcripts uploaded into N-Vivo (QSR International) software and analysed using Braun and Clarke’s model of thematic analysis [37]. Authentication of key themes was undertaken by discussion and consensus with the research fellow/ECHO clinic administrator (BDWJ) and the principal investigator/ECHO clinic facilitator (CP).

## Results

The numbers and types of HCPs participating in each of the five ECHO clinics are detailed in Table 3. HCPs participated in one or more clinic(s); all were invited to complete post-ECHO evaluations.

**Table 3:** Characteristics of healthcare professionals participating in each of the TEAM Pain AD teleECHO clinics

Healthcare professional	Area of clinical practice	Setting of clinical practice	ECHO 1 (N)	ECHO 2 (N)	ECHO 3 (N)	ECHO 4 (N)	ECHO 5 (N)
HCA	Nursing home	Nursing home	3	0	1	0	0
Nurse	Dementia	Secondary care	1	2	3	2	2
Nurse	Nursing home	Nursing home	1	1	4	3	1
Nurse	Nurse Education	Secondary care	0	1	0	0	0
Nurse	Mental Health	Secondary care	0	1	0	6	3
Nurse	Palliative care	Hospice	6	3	4	5	7
Nurse	Palliative care	Secondary care	0	0	1	0	0
Nurse	Pain	Secondary care	1	0	1	0	0
Occupational Therapist	Dementia	Secondary care	0	1	0	1	0
Pharmacist	Pharmacy and Medicines Management	Health and Social Care Board	1	2	0	0	0
Physician	General Practice	Hospice	0	0	1	0	1
Physician	General Practice	Primary care	0	3	3	0	0
Physician	Pain	Secondary care	1	0	1	0	0
Physician	Palliative care	Hospice	2	0	1	1	0
Physician	Palliative care	Secondary care	1	0	0	0	0
Physician	Geriatrics	Secondary care	0	1	0	2	1
Physician	Psychiatry	Secondary care	0	3	1	3	3
Social worker	Mental Health	Secondary care	0	0	0	1	0
Total			17	18	21	24	18

*HCA* Healthcare assistant

### Pre- and post-ECHO evaluations

Eighteen HCPs (seven physicians, ten nurses and one HCA) completed the respective pre-ECHO knowledge and efficacy evaluations, and twenty completed the post-ECHO evaluations (ten physicians and ten nurses). Responses to the evaluations are detailed in Additional files 1, 2, 3, 4 and 5.

Physician pre-ECHO questionnaire responses (Additional file 1) suggested that there were some areas in which some respondents lacked confidence, indicated by responses in the Strongly Disagree, Disagree, and Neither Agree nor Disagree categories. These included: confidence in prescribing analgesia for administration via syringe driver; intravenous route or transdermal routes; clinical knowledge of pain assessment and management; clinical self-efficacy; and use of best practice approaches in pain assessment and management. In the post-ECHO evaluations (Additional file 1), no respondents selected Strongly Disagree for any statement, and there were marked reductions in the numbers who chose Disagree and Neither Agree nor Disagree options, with the majority now selecting Agree or Strongly Agree for each statement. The post-ECHO evaluation (Additional file 4) also demonstrated the perceived utility of the teleECHO clinics; the majority of respondents (70% or more) agreed or strongly agreed to each of the statements in this evaluation which considered development of knowledge and skills in pain assessment and management, application of knowledge gained through the clinics, benefit to clinical practice, the value of case-based learning and didactic teaching, and the value of continued clinics.

Nurse pre-ECHO evaluation responses (Additional file 2) indicated that the majority of nurses felt confident reporting pain, assessing treatment response to analgesia, suggesting alternative formulations if the oral route was unavailable, and in discussing cases of unresolved pain, as evidenced by most respondents selecting Agree or Strongly Agree for these statements. There was greater uncertainty, demonstrated by respondents selecting Disagree or Neither Agree nor Disagree in relation to feeling confident in the following areas: recognising and assessing pain in patients with advanced dementia nearing the end of life; differentiating behavioural indicators of pain from BPSD; recognising and managing breakthrough pain; clinical knowledge and self-efficacy; and using best practice approaches to pain assessment and pain management. Similar to physicians, there were marked reductions in the numbers who chose Disagree and Neither Agree nor Disagree options in the post-ECHO evaluation, with the majority now selecting Agree or Strongly Agree for each statement (Additional file 2). The post-ECHO evaluation also demonstrated the perceived utility of the teleECHO clinics for nurses (Additional file 5); the majority of respondents agreed or strongly agreed that they had developed their clinical knowledge and skills in pain assessment and pain management, that they had applied the knowledge learnt and taught other staff what they had learned, that access to expertise had benefitted their clinical practice, and that case-based discussion and didactic sessions were effective ways to develop clinical knowledge and skills. They also indicated that they would support continued clinics for this and other clinical issues. The only area in which opinions differed was whether clinics specifically aimed at nurses would be beneficial, with similar proportions of respondents agreeing or disagreeing with this statement.

The HCA who completed the pre-ECHO evaluation of knowledge and self-efficacy reported that he/she was confident in recognising and reporting pain, differentiating between pain and non-pain related challenging behaviour, and discussing pain assessment and management with doctors and nurses (Additional file 3).

Statistical analysis of physician and nurse scores for knowledge and self-efficacy in pain assessment and management in advanced and end-stage dementia demonstrated that overall knowledge and efficacy scores were significantly higher post-ECHO than pre-ECHO (*p* = 0.014 and *p* = 0.035 for physicians and nurses respectively; Table 4). As no HCAs completed the post-ECHO evaluation, it was not possible to determine a knowledge and efficacy score for HCAs following participation in the clinics or to compare pre- and post-ECHO scores.

**Table 4:** Knowledge and self-efficacy results

HCP type	Possible score range	Pre-ECHO knowledge and self-efficacy score		Post-ECHO knowledge and self-efficacy score		*p*-value
		Mean	SD	Mean	SD	
Physician	14–70	41.4 *n* = 7	10.6	55.8 *n* = 10	10.2	0.014^*^
Nurse	11–55	37.9 *n* = 10	6.5	44.8 *n* = 10	7.0	0.035^*^
HCA	7–35	28.0 *n* = 1	-	-	-	-

*HCA* Healthcare assistant, *HCP* Healthcare professional

^*^Mann-Whitney U-test

- Not available

### Focus group interviews

Seven individuals participated in two focus groups (three in Focus Group 1 and four in Focus Group 2). Participants in Focus Group 1 were specialist nurses (dementia *n* = 1, hospice *n* = 2). Participants in Focus Group 2 included a GP, a consultant physician (geriatrics) and two specialist hospice nurses. Four core themes emerged and are presented below.

### Theme 1: Knowledge and skills development and dissemination

Participants reported that they had gained new clinical knowledge and skills through participation in the ECHO clinics. In most cases, this was a result of participating in the case discussions in which knowledge and skills were freely exchanged among the experts at the hub and other participants dialling in from the spokes.

*I liked having access to people with—with specialist knowledge and experience that was very helpful (GP4, FG2).*

In most cases, knowledge and skills development pertained to novel, holistic or alternative approaches to care, behavioural management of patients with dementia, pharmacological and non-pharmacological interventions for pain management, aspects of pain assessment and ethical and professional practice issues. Most participants believed they had applied these knowledge and skills to their own patients, whilst others reported disseminating these to their clinical teams. Those who had submitted a patient case for discussion reported that they had adopted the treatment recommendations resulting in improvements to the patient’s care and strengthening of the relationship between the clinical team and the patient’s family, and had trained other staff following the transfer of the patient to another care setting. Most respondents had actively contributed to the case discussions and expressed that having this opportunity was essential to their learning and development. They felt that the combination of access to a panel of experts and being able to participate interactively made ECHO a unique learning experience both professionally and personally.

*Access to all the professionals and even when the cases were being discussed and that, even though they were very professional they were sort of informal and it was a very comfortable way of discussing things, I actually enjoyed it (Hospice nurse 6, FG2).*

Some participants reported that whilst participation may not have resulted in new skills and knowledge development, they had felt reassured that their approaches to complex and challenging patient care were in line with best practice and with what the expert panel were practising themselves.

*….sometimes it’s just about reassuring staff they’re doing the right thing. I think that comes through in some of the cases, um, you’re doing everything you can and that’s sometimes good that reassurance and that’s good with their own discipline, but certainly for knowledge (Dementia nurse 1, FG1).*

All participants agreed that hearing the experiences of the other ECHO participants allowed them to reframe how they perceived their own difficulties, contextualizing them as a natural by-product of caring for a complex patient population, rather than an indicator of personal or professional failure. This reassured participants and increased professional and self-confidence, morale, and motivation. For many, this was a significant benefit of participating in ECHO.

### Theme 2: Protected time

Participants reported that a significant benefit of the ECHO model was the ability to join clinics from their own workplaces, eliminating the need for travel, expenses and time out of clinical practice.

*The convenience of, you know, being able to …. dial in from … my laptop in work is very helpful….. for the two of us contributing here today up in [Trust], having to get down on a weekly basis to something in Belfast you know is not … feasible (Geriatrician 7, FG2).*

This was particularly important considering the geographical spread of participants who took part in this study; one participant, however, noted that this convenience was also a ‘double-edged sword’ in that being physically present in the office or building encouraged staff to call them away to attend to clinical matters on the ward.

Many participants reported that protected time was required to allow staff to participate in ECHO clinics. Some recognised that this was easier to achieve in some settings (e.g. hospice) than others (e.g. primary and secondary care). Respondents strongly believed that ECHO clinics needed to be planned well in advance and appropriately advertised, allowing staff rotas to be adjusted to ensure sufficient cover and thereby minimise the impact of staff absence from the wards/clinics for the duration of ECHO sessions. Participants agreed that individual work plans needed to reflect participation in ECHO clinics as protected time to allow staff to participate uninterrupted and to prepare case studies.

*It just needs to be planned you know …… certainly the setting we’re in here which is in a day hospice setting it’s easier I know than in [hospital setting] or in a GP setting it’s so much more difficult to have protected time, and it is I suppose making it explicit at the beginning that protected time is needed in some way so that any individual taking part can have a commitment from their colleagues that they will have protected time…and that’s always difficult. (GP4, FG2).*

### Theme 3: Areas for improvement

Participants noted some difficulties experienced with the submission of case studies. It was tentatively suggested that the novel format of ECHO which involved a diverse audience of clinical professionals across trusts, networks and regions may have contributed to reticence among participants to submit a case study in which the challenges experienced by the submitting team would be widely exposed. Some noted this resulted in late submission and dissemination of case materials leaving little time for review and preparation ahead of clinics. It was also reported that case submissions took time to prepare and write; therefore, sufficient time and opportunity were required to allow staff to complete this.

*That was just a bit of typical ……. reticence to put yourselves forward, put your head above the parapet, you know, to put a case out there but once the cases were there I think that led … to*. *…. good back and forth conversation between the group….. I guess it’s in terms of how to encourage folk to, you know, to put the cases forward maybe a bit more in advance you know for fuller preparation for the sessions. (Geriatrician 7, FG2).*

Participants suggested that future ECHOs would need to consider an alternative approach to obtaining case study submissions well in advance of clinics. Participants commented that occasional technical glitches resulted in sound and video quality impairment and delays logging in to clinics. It was also noted that delays at the start of clinics reduced time for case discussion and on one occasion it was felt that the submitting team had been left without a clear resolution or treatment plan. However, despite the technical issues experienced, one participant reported that the technology was more efficient than existing videoconferencing facilities in their organisation and that accessing clinics had been easy and quick.

### Theme 4: The future of ECHO

Most participants strongly welcomed further ECHO clinics in dementia, pain and other chronic conditions. All agreed that the model was suitable for addressing the learning needs of HCPs through a combination of didactic training by appropriately qualified and experienced clinical staff and opportunity for case discussion. All reported that the most significant strength of the ECHO model lay in its multidisciplinary, inclusive approach which created and fostered a sense of community.

*I like … all the different multidisciplinary teams because they bring different information you know because it gives you confidence listening to them and you know you can speak to them (Hospice nurse 3, FG1).*

Participants did not see any benefit in holding discipline-specific ECHO clinics (e.g. those to which only nurses or physicians etc. attended) but did believe that ECHO programmes in dementia could be broadened out so that they included other aspects of care rather than a specific focus on one area (e.g. pain). Interconnectivity among frontline and allied health professionals was perceived as the cornerstone of dementia care from which gold standards could be achieved.

*I think absolutely broadened out and encouraged …. we all work in areas where knowledge is constantly evolving, you know, and … where the challenges that we face are changing and I suppose in any world of healthcare every person brings a unique story and unique talent so you know we’re all learning all the time and it’s a great format for learning so I would certainly be very supportive of the approach (GP4, FG2).*

Additionally, developing cross-specialty networks which bridged primary, secondary, nursing home, community and hospice care across Health and Social Care (HSC) trusts and geographical regions allowed participants to gain perspective on the nature of dementia care across Northern Ireland.

*Because we use it within our teams and we’re across trusts, it allows us to explore even lack of equity across trusts and services and things like that so it’s always good to hear what other trusts and services are doing which ECHO will allow you to do. (Hospice nurse 2, FG1).*

Most participants reported that the bigger picture perspective allowed them to see themselves as part of a community of professionals facing the challenges of managing and caring for a complex patient population; this was important for reducing feelings of professional isolation and maintaining morale and motivation. Participants commented on the potential of ECHO to inform and improve the delivery of clinical education and ongoing professional development.

## Discussion

The evaluation of the TEAM Pain AD teleECHO clinics, based on the findings from the pre-, and post-ECHO evaluations and the focus group discussions, was largely very positive. Physician pre-ECHO questionnaire responses suggested that some respondents lacked confidence in prescribing analgesia for administration via syringe driver, intravenous or transdermal routes, clinical knowledge of pain assessment and management, clinical self-efficacy, and use of best practice approaches in pain assessment and management. Post-ECHO evaluations suggested that after clinic participation, respondents felt more confident in prescribing medications for administration via routes other than orally, in their clinical knowledge and self-efficacy and in use of best practice approaches. Most physician respondents reported development of their knowledge and skills in pain assessment and management, application of knowledge gained through the clinics, benefit to their clinical practice, the value of case-based learning and didactic teaching, and the value of continued clinics. Similarly, prior to undertaking the TEAM Pain AD teleECHO clinics, some nurses expressed a lack of confidence in recognising and assessing pain, differentiating behavioural indicators of pain from BPSD, recognising and managing breakthrough pain, clinical knowledge and self-efficacy, and using best practice approaches to pain assessment and pain management. Post-ECHO evaluations suggested that confidence in these areas had improved. Many respondents reported that they had developed their clinical knowledge and skills in pain assessment and pain management, applied the knowledge learnt and taught other staff what they had learned, and that access to expertise had benefitted their clinical practice. They felt that case-based discussion and didactic sessions were effective ways to develop clinical knowledge and skills and indicated support for continued clinics for this and other clinical issues. Analysis of physician and nurse scores for knowledge and self-efficacy suggest increased confidence in relation to knowledge and self-efficacy in post-ECHO evaluations compared to the pre-ECHO survey. These findings are similar to results from other studies that have used Project ECHO for palliative care interventions [32, 38], HIV [39], chronic pain [31], complex disease management [40], hypertension [27], diabetes [41] and for knowledge networks across a range of clinical areas (diabetes, optometry, palliative care in nursing homes, dermatology, and support for carers of patients with palliative care needs) [33]. The focus groups confirmed these findings, with participants reporting gaining new knowledge and skills, or where new skills and knowledge were not developed, reassurance that they were using approaches in line with best practice and with what the experts were practising themselves. The focus groups also reported that a further benefit of the ECHO© model was the ability to join clinics without having to leave the workplace, eliminating the need for travel, expense and significant periods of time away from clinical practice. However, protected time was crucial to facilitate clinic participation. Areas in which improvements were required included submission of case studies in a timely manner for dissemination to all participants well in advance of the clinic, and improved sound and video quality. However, technical issues were not sufficient to discourage participation in future clinics. Technical issues, in particular internet connectivity and bandwidth, have been identified as problematic by others [32]; however, similar to our study, these issues were not at a level to prevent the vast majority of participants from being willing to recommend ECHO© to others. The potential of ECHO© to inform and improve delivery of clinical education and continuing professional development was recognised, with the most significant strength of the model reported to be its multidisciplinary, inclusive approach which created and fostered a sense of community. This emphasis on a “community of learners” affirms the Community of Practice Theory, which emphasises the importance of learning through continuous participation in a collaborative community consisting of peer learners and expert individuals, as a foundation of the ECHO© model [42], and which has been reported in other studies [22, 33].

The pre- and post-ECHO evaluations and focus group interviews suggest the value of the Project ECHO© model in enhancing HCP confidence in knowledge and self-efficacy in assessing and managing pain for people with advanced dementia, and the potential for this type of educational intervention in other clinical areas. The data suggest increased confidence in knowledge and self-efficacy after participation in the teleECHO clinics; focus group participants expressed a desire for confirmation of their proposed treatment; and reported that receiving support from other specialties and knowing they were ‘on the right track’ with prescribing and treatment increased their confidence and job satisfaction. Further, the post-ECHO physician and nurse evaluations demonstrated the perceived utility of the clinics in development of clinical knowledge and skills in pain assessment and management, application of knowledge gained, benefit to clinical practice, the value of case-based learning and didactic teaching, and indicated continued support for pain clinics and for other clinical issues. The adoption of this model of training and education, not only in the clinical area of pain in dementia, but also in other clinical areas is therefore recommended. The ECHO© model should continue to be developed and evaluated in terms of its impact, not only on HCP knowledge and self-efficacy, but also on service delivery and patient outcomes. Work is required to enhance response rates in future evaluations and to ensure that future ECHO© networks meet the needs of the population for whom they are intended. This should address minor technological issues to enhance sound and video quality and connectivity, and to facilitate access from some sites currently unable to connect due to security policies.

Our findings must be interpreted in the context of the limitations we experienced, both in the delivery of the teleECHO© clinics and in their evaluation. Firstly, despite having approximately five weeks between the pre-ECHO© workshop (at which the curriculum, times and dates of the clinics were decided) and the first teleECHO© clinic, it was extremely difficult to get patient cases. Participants were reticent to put forward cases, and this resulted in circulation of cases on the day before or the day of the clinic, which did not allow sufficient time for participants to familiarise themselves with the case before the start of the clinic. Secondly, there were some technical issues due to poor sound quality and unstable internet connections. Thirdly, it was not possible for the Zoom teleconferencing and camera equipment and software to be approved on computers for one HSC Trust, meaning that the firewall prevented participation of HCPs from that Trust. Furthermore, it was not possible to administer the knowledge and self-efficacy evaluation to respondents on three occasions, in pre-, post- and retrospective-pre teleECHO evaluations, due to respondent fatigue. The aim of the retrospective-pre evaluation is to reflect back and rate knowledge and self-efficacy before participation in the ECHO clinics with the benefit of hindsight [43, 44]; we were not able to collect these data. Other studies have reported similar difficulties in low evaluation response rates [33]. A recent systematic review revealed similar limitations reported in 39 published studies spanning 17 health conditions and called for further exploration of the barriers to implementing Project ECHO© in clinical practice [45]. Additionally, for the physician and nurse pre- and post-ECHO evaluations, it was not possible to compare changes in individuals’ responses between the pre- and retro-pre evaluations as respondents completed evaluation questionnaires anonymously. It is therefore possible that the improvement in knowledge and self-efficacy observed may be due to differences in the participants, rather than participation in the clinics. However, analysis of the focus group evaluations suggest that this enhanced knowledge and self-efficacy is likely to be associated with participation in the teleECHO clinics. A further limitation was that only one HCA completed the pre-ECHO evaluation and no HCAs completed the post-ECHO evaluation, despite assurances from the research team regarding anonymity and confidentiality. It was therefore not possible to examine knowledge and self-efficacy scores pre- and post-ECHO clinic participation for these HCPs. Reasons for this may include a lack of engagement with the process of evaluation or a feeling that it was not applicable, or a fear that if they are deemed not to be delivering best practice, this may be used against them. Furthermore, HCAs do not routinely have regular access to computers, with the exception of undertaking mandatory online training, and this may have acted as a barrier to completion of online evaluation of the TEAM Pain AD teleECHO© clinics. A further limitation with regard to the focus groups relates to the small numbers within each group (three participants in one focus group and four in the other). Finally, the direct impact of the TEAM Pain AD teleECHO© clinics on patient and/or carer outcomes were not examined in this study.

Project ECHO© has demonstrated early positive evidence for improving knowledge and skills among care providers; however, a need for further evaluation of patient outcomes using validated outcome measures and exploration of the limitations associated with its evaluation has been highlighted [45]. This is likely to be facilitated by the recent passing of the Expanding Capacity for Health Outcomes (ECHO) Act in the United States, the country in which Project ECHO© was originally developed [46], which is anticipated to result in the adoption of Project ECHO© as the national model for provision of rural telehealth care provision in the United States. This lends further support to the development of Project ECHO© telementoring clinics for HCP education and training internationally.

## Conclusion

The results from this study support the use of Project ECHO© telementoring clinics for HCP education and training in pain assessment and management in advanced and end-stage dementia. They suggest a positive impact on knowledge and self-efficacy and highlight the value of a cross-specialty network of practice which bridges discipline/HCP type, primary, secondary, community and hospice care settings, and geographical areas. Further development of ECHO© services in pain assessment and management in dementia, and in other clinical areas, has the potential to support the delivery of high-quality care for complex patient populations.

## Additional files

Additional file 1: **Table S1.** Pre- and post-teleECHO knowledge and self-efficacy evaluation responses: physicians. (DOCX 14 kb)

Additional file 2: **Table S2.** Pre- and post-teleECHO knowledge and self-efficacy evaluation responses: nurses. (DOCX 17 kb)

Additional file 3: **Table S3.** Pre-ECHO knowledge and self-efficacy evaluation responses: healthcare assistants. (DOCX 19 kb)

Additional file 4: **Table S4.** Post-ECHO evaluation responses: physicians. (DOCX 14 kb)

Additional file 5: **Table S5.** Post-ECHO evaluation responses: nurses. (DOCX 14 kb)

## Abbreviations

ECHO©: Extension for Community Healthcare Outcomes
FG: Focus Group
HCA: Healthcare assistant
HCP: Healthcare professional
HSC: Health and social care
NI: Northern Ireland
PMG: Project management group
TEAM Pain AD: Telementoring to Enhance Assessment and Management of Pain in Advanced Dementia
UK: United Kingdom
