# Supplementary material for: Evaluation of the impact of telementoring using ECHO© technology on healthcare professionals’ knowledge and self-efficacy in assessing and managing pain for people with advanced dementia nearing the end of life
Source: BMC Health Serv Res. 2018 Apr 2;18:228. doi: 10.1186/s12913-018-3032-y (PMC5879835; doi:10.1186/s12913-018-3032-y)
Supplement: Supplementary file 1 — Table S1. Pre- and post-teleECHO knowledge and self-efficacy evaluation responses: physicians. (DOCX 14 kb) [file 12913_2018_3032_MOESM1_ESM.docx]

**Additional file 1: Table S1. Pre- and post teleECHO knowledge and self-efficacy evaluations: physicians**

| **Knowledge and efficacy evaluation statement** | **Strongly Disagree** | **Strongly Disagree** | **Disagree** | **Disagree** | **Neither Agree nor Disagree** | **Neither Agree nor Disagree** | **Agree** | **Agree** | **Strongly Agree** | **Strongly Agree** |
| --- | --- | --- | --- | --- | --- | --- | --- | --- | --- | --- |
|  | **Pre-ECHO** | **Post-ECHO** | **Pre-ECHO** | **Post-ECHO** | **Pre-ECHO** | **Post-ECHO** | **Pre-ECHO** | **Post-ECHO** | **Pre-ECHO** | **Post-ECHO** |
| 1. I feel confident **recognising and assessing pain** in patients with advanced dementia nearing the end of life | 0 (0) | 0 (0) | 1 (14.3) | 0 (0) | 1 (14.3) | 1 (10) | 5 (71.4) | 6 (60) | 0 (0) | 3 (30) |
| 2. I feel confident establishing a pain diagnosis for patients with advanced dementia nearing the end of life | 0 (0) | 0 (0) | 1 (14.3) | 0 (0) | 2 (28.6) | 0 (0) | 4 (57.1) | 8 (80) | 0 (0) | 2 (20) |
| 3. I feel confident differentiating the behavioural indicators of pain from behavioural and psychological symptoms of dementia in patients with advanced dementia nearing the end of life | 0 (0) | 0 (0) | 1 (14.3) | 0 (0) | 4 (57.1) | 4 (40) | 2 (28.6) | 4 (40) | 0 (0) | 2 (20) |
| 4. I feel confident **prescribing for pain** in patients with advanced dementia nearing the end of life | 0 (0) | 0 (0) | 2 (28.6) | 0 (0) | 2 (28.6) | 1 (10) | 3 (42.9) | 6 (60) | 0 (0) | 3 (30) |
| 5. I feel confident prescribing for and managing **breakthrough pain** in people with advanced dementia nearing the end of life | 0 (0) | 0 (0) | 2 (28.6) | 0 (0) | 3 (42.9) | 1 (10) | 2 (28.6) | 6 (60) | 0 (0) | 3 (30) |
| 6. I feel confident assessing treatment response to analgesics in patients with advanced dementia who are nearing the end of life | 0 (0) | 0 (0) | 1 (14.3) | 0 (0) | 4 (57.1) | 1 (10) | 2 (28.6) | 7 (70) | 0 (0) | 2 (20) |
| 7. I feel confident prescribing analgesia for administration by **syringe driver** in advanced dementia at end of life | 1 (14.3) | 0 (0) | 3 (42.9) | 0 (0) | 1 (14.3) | 1 (10) | 2 (28.6) | 6 (60) | 0 (0) | 3 (30) |
| 8. I feel confident prescribing analgesia for **intravenous (IV) administration** in advanced dementia at end of life | 2 (28.6) | 2 (20) | 2 (28.6) | 1 (10) | 2 (28.6) | 3 (30) | 1 (14.3) | 2 (20) | 0 (0) | 2 (20) |
| 9. I feel confident prescribing **transdermal analgesics** in advanced dementia at end of life | 0 (0) | 0 (0) | 4 (57.1) | 0 (0) | 0 (0) | 0 (0) | 3 (42.9) | 6 (60) | 0 (0) | 4 (40) |
| 10. I feel confident prescribing analgesia for **subcutaneous administration** in advanced dementia at end of life | 1 (14.3) | 0 (0) | 3 (42.9) | 0 (0) | 0 (0) | 0 (0) | 3 (42.9) | 7 (70) | 0 (0) | 3 (30) |
| 11. I feel confident in my **clinical knowledge** of pain assessment and management in patients with advanced dementia nearing the end of life | 1 (14.3) | 0 (0) | 0 (0) | 0 (0) | 3 (42.9) | 1 (10) | 3 (42.9) | 7 (70) | 0 (0) | 2 (20) |
| 12. I feel confident in **my clinical self-efficacy** in the assessment and management of pain in patients with advanced dementia nearing the end of life | 0 (0) | 0 (0) | 3 (42.9) | 0 (0) | 2 (28.6) | 1 (10) | 2 (28.6) | 7 (70) | 0 (0) | 2 (20) |
| 13. I feel confident I am using best-practice approaches to **pain assessment** in patients with advanced dementia nearing the end of life | 0 (0) | 0 (0) | 3 (42.9) | 1 (10) | 3 (42.9) | 0 (0) | 1 (14.3) | 7 (70) | 0 (0) | 2 (20) |
| 14. I feel confident I am using best practice approaches to pain management in patients with advanced dementia nearing the end of life | 0 (0) | 0 (0) | 3 (42.9) | 1 (10) | 2 (28.6) | 0 (0) | 2 (28.6) | 7 (70) | 0 (0) | 2 (20) |
