# Supplementary material for: Evaluation of the impact of telementoring using ECHO© technology on healthcare professionals’ knowledge and self-efficacy in assessing and managing pain for people with advanced dementia nearing the end of life
Source: BMC Health Serv Res. 2018 Apr 2;18:228. doi: 10.1186/s12913-018-3032-y (PMC5879835; doi:10.1186/s12913-018-3032-y)
Supplement: Supplementary file 2 — Table S2. Pre- and post-teleECHO knowledge and self-efficacy evaluation responses: nurses. (DOCX 17 kb) [file 12913_2018_3032_MOESM2_ESM.docx]

**Additional file 2: Table S2. Pre- and post-teleECHO knowledge and self-efficacy evaluations: nurses**

| **Knowledge and efficacy evaluation statement** | **Strongly Disagree** | **Strongly Disagree** | **Disagree** | **Disagree** | **Neither Agree nor Disagree** | **Neither Agree nor Disagree** | **Agree** | **Agree** | **Strongly Agree** | **Strongly Agree** |
| --- | --- | --- | --- | --- | --- | --- | --- | --- | --- | --- |
|  | **Pre-ECHO** | **Post-ECHO** | **Pre-ECHO** | **Post-ECHO** | **Pre-ECHO** | **Post-ECHO** | **Pre-ECHO** | **Post-ECHO** | **Pre-ECHO** | **Post-ECHO** |
| 1. I feel confident **recognising and assessing pain** in patients with advanced dementia nearing the end of life | 0 (0) | 0 (0) | 1 (10) | 0 (0) | 4 (40) | 5 (41.7) | 4 (40) | 4 (33.3) | 1 (10) | 3 (25) |
| 2. I feel confident reporting pain in patients with advanced dementia nearing the end of life | 0 (0) | 0 (0) | 1 (10) | 0 (0) | 2 (20) | 2 (16.7) | 6 (60) | 7 (58.3) | 1 (10) | 3 (25) |
| 3. I feel confident **differentiating the behavioural indicators of pain** from behavioural and psychological symptoms of dementia in patients with advanced dementia nearing the end of life | 0 (0) | 0 (0) | 3 (30) | 1 (8.3) | 2 (20) | 3 (25) | 4 (40) | 6 (50) | 1 (10) | 2 (16.7) |
| 4.I feel confident **administering** analgesia **by syringe driver** to patients with advanced dementia nearing the end of life* | 0 (0) | 0 (0) | 0 (0) | 0 (0) | 0 (0) | 0 (0) | 0 (0) | 0 (0) | 2 (100)^a^ | 2(100)^b^ |
| 5. I feel confident **administering** analgesia by **intravenous routes** to patients with advanced dementia nearing the end of life* | 0 (0) | 0 (0) | 0 (0) | 0 (0) | 0 (0) | 0 (0) | 2 (100)^a^ | 0 (0) | 0 (0) | 1(100)^b^ |

| 6. I feel confident assessing treatment response to analgesics in patients with advanced dementia who are nearing the end of life | 0 (0) | 0 (0) | 0 (0) | 0 (0) | 2 (20) | 3 (30) | 8 (80) | 4 (40) | 0 (0) | 3 (30) |
| --- | --- | --- | --- | --- | --- | --- | --- | --- | --- | --- |
| 7. I feel confident suggesting alternative formulations of analgesia when oral route is unavailable | 0 (0) | 0 (0) | 2 (20) | 0 (0) | 0 (0) | 1 (10) | 7 (70) | 7 (70) | 1 (10) | 2 (20) |
| 8. I feel confident recognising and managing **breakthrough pain** in people with advanced dementia nearing the end of life | 0 (0) | 0 (0) | 3 (30) | 0 (0) | 2 (20) | 3 (30) | 5 (50) | 6 (60) | 0 (0) | 1 (10) |
| 9. I feel confident discussing cases of unresolved pain following administration of analgesia with doctors for patients with advanced dementia nearing the end of life | 0 (0) | 0 (0) | 2 (20) | 0 (0) | 2 (20) | 2 (20) | 5 (50) | 4 (40) | 1 (10) | 4 (40) |
| 10. I feel confident in my **clinical knowledge** of pain assessment and management in patients with advanced dementia nearing the end of life | 0 (0) | 0 (0) | 2 (20) | 1 (10) | 3 (30) | 0 (0) | 5 (50) | 5 (50) | 0 (0) | 4 (40) |
| 11. I feel confident in **my clinical self-efficacy** in the assessment and management of pain in patients with advanced dementia nearing the end of life | 0 (0) | 0 (0) | 3 (30) | 1 (10) | 2 (20) | 1 (10) | 5 (50) | 4 (40) | 0 (0) | 4 (40) |
| 12. I feel confident I am using best practice approaches to pain assessment in patients with advanced dementia nearing the end of life | 0 (0) | 0 (0) | 1 (10) | 1 (10) | 5 (50) | 0 (0) | 4 (40) | 5 (50) | 0 (0) | 4 (40) |
| 13. I feel confident I am using best practice approaches to pain management in patients with advanced dementia nearing the end of life | 0 (0) | 0 (0) | 0 (0) | 0 (0) | 6 (60) | 2 (20) | 4 (40) | 4 (40) | 0 (0) | 4 (40) |

^a^ 2 respondents administered analgesia to patients with advanced dementia nearing the end of life via syringe driver or intravenous routes and could therefore respond to statements 4 and 5 respectively in the Pre-ECHO evaluation

^b^ 2 respondents administered analgesia to patients with advanced dementia nearing the end of life via syringe driver, and one administered analgesia via intravenous routes and could therefore respond to statements 4 and 5 respectively in the Post-ECHO evaluation
