# Supplementary material for: Evaluation of the impact of telementoring using ECHO© technology on healthcare professionals’ knowledge and self-efficacy in assessing and managing pain for people with advanced dementia nearing the end of life
Source: BMC Health Serv Res. 2018 Apr 2;18:228. doi: 10.1186/s12913-018-3032-y (PMC5879835; doi:10.1186/s12913-018-3032-y)
Supplement: Supplementary file 3 — Table S3. Pre-ECHO knowledge and self-efficacy evaluation responses: healthcare assistants. (DOCX 19 kb) [file 12913_2018_3032_MOESM3_ESM.docx]

**Additional file 3: Table S3. Pre-ECHO knowledge and self-efficacy questionnaire responses: HCAs**

| **Knowledge and efficacy evaluation statement** | **Number (%) of respondents who selected** | | | | |
| --- | --- | --- | --- | --- | --- |
|  | **Strongly**  **Disagree** | **Disagree** | **Neither Agree nor Disagree** | **Agree** | **Strongly**  **Agree** |
| 1. I feel confident **recognising pain** in patients with advanced dementia nearing the end of life | 0 | 0 | 0 | 1 (100) | 0 |
| 2. I feel confident **reporting pain** in patients with advanced dementia nearing the end of life | 0 | 0 | 0 | 1 (100) | 0 |
| 3. I feel confident **identifying pain** from challenging behaviour in patients with advanced dementia nearing the end of life | 0 | 0 | 0 | 1 (100) | 0 |
| 4. I feel confident discussing **pain assessment** **with doctors** | 0 | 0 | 0 | 1 (100) | 0 |
| 5. I feel confident discussing **pain management with doctors** | 0 | 0 | 0 | 1 (100) | 0 |
| 6. I feel confident discussing **pain assessment with nurses** | 0 | 0 | 0 | 1 (100) | 0 |
| 7. I feel confident discussing **pain management** **with nurses** | 0 | 0 | 0 | 1 (100) | 0 |
