# Supplementary material for: Evaluation of the impact of telementoring using ECHO© technology on healthcare professionals’ knowledge and self-efficacy in assessing and managing pain for people with advanced dementia nearing the end of life
Source: BMC Health Serv Res. 2018 Apr 2;18:228. doi: 10.1186/s12913-018-3032-y (PMC5879835; doi:10.1186/s12913-018-3032-y)
Supplement: Supplementary file 4 — Table S4. Post-ECHO evaluation responses: physicians. (DOCX 14 kb) [file 12913_2018_3032_MOESM4_ESM.docx]

**Additional File 4: Table S4. Post-ECHO evaluation: physicians**

|  | **Number (%) of respondents selecting** | | | | |
| --- | --- | --- | --- | --- | --- |
|  | **Strongly**  **Disagree** | **Disagree** | **Neither Agree nor Disagree** | **Agree** | **Strongly**  **Agree** |
| 1. Participation in the TEAM Pain AD teleECHO clinics has developed my clinical knowledge in pain assessment in advanced dementia | **0 (0)** | **0 (0)** | **2 (20)** | **5 (50)** | **3 (30)** |
| 2. Participation in the TEAM Pain AD teleECHO clinics has developed my clinical skills in pain assessment in advanced dementia | **0 (0)** | **0 (0)** | **2 (20)** | **6 (60)** | **2 (20)** |
| 3. Participation in the TEAM Pain AD teleECHO clinics has developed my clinical knowledge in pain management in advanced dementia | **0 (0)** | **0 (0)** | **0 (0)** | **6 (60)** | **2 (20)** |
| 4. Participation in the TEAM Pain AD teleECHO clinics has developed my clinical skills in pain management in advanced dementia | **0 (0)** | **0 (0)** | **2 (20)** | **7 (70)** | **1 (10)** |
| 5. Did you present a patient case at a TEAM Pain AD teleECHO clinic?  IF YES:  I am confident/comfortable presenting patient cases during TEAM Pain AD teleECHO clinics  Presenting a patient case in the TEAM Pain AD teleECHO clinics benefitted the patient in my care  IF NO:  I would be confident/ comfortable presenting patient cases during TEAM Pain AD teleECHO clinics  I learned from providers who present their patient cases during TEAM Pain AD teleECHO clinics | **0 (0)**  **0 (0)**  **0 (0)**  **0 (0)** | **0 (0)**  **0 (0)**  **0 (0)**  **0 (0)** | **0 (0)**  **0 (0)**  **1 (16.7)**  **0 (0)** | **4 (100)**  **2(50)**  **3 (50)**  **5(83.3)** | **0 (0)**  **2(50)**  **2 (33.3)**  **1(16.7)** |
| 6. I apply knowledge learned in TEAM Pain AD teleECHO clinics to other patients who have similar symptoms in my care | 0 (0) | 0 (0) | 3 (30) | 6 (60) | 1 (10) |
| 7. I teach other clinical staff what I have learned in TEAM Pain AD teleECHO clinics | 0 (0) | 0 (0) | 3 (30) | 6 (60) | 1 (10) |
| 8. Access to specialist expertise and consultation is an important area of need for me and my care staff team | 0 (0) | 0 (0) | 0 (0) | 7 (70) | 3 (30) |
| 9. Collaboration with specialists and physicians from other specialties has been a benefit to my clinical practice | 0 (0) | 0 (0) | 0 (0) | 7 (70) | 3 (30) |
| 10. Access to expertise in **pharmacology** has benefitted my clinical knowledge and practice | 0 (0) | 0 (0) | 0 (0) | 6 (60) | 4 (40) |
| 11. Access to expertise in **behaviour and mental health** has benefitted my clinical knowledge and practice | 0 (0) | 0 (0) | 0 (0) | 5 (50) | 5 (50) |
| 12. TEAM Pain AD teleECHO clinics have improved the way that health professionals **communicate** with each other **about pain** in patients with advanced dementia nearing the end of life | 0 (0) | 0 (0) | 2 (20) | 4 (40) | 4 (40) |
| 13. Learning about complex chronic disease through participation in TEAM Pain AD teleECHO clinics is an effective way to enhance clinical knowledge and skills | 0 (0) | 0 (0) | 0 (0) | 6 (60) | 4 (40) |
| 14. Case-based learning as the focus for discussion is an impactful way of learning | 0 (0) | 0 (0) | 0 (0) | 4 (40) | 6 (60) |
| 15. Didactic sessions during TEAM Pain AD teleECHO clinics were an effective way for me to develop my clinical knowledge and skills | 0 (0) | 0 (0) | 1 (10) | 6 (60) | 3 (30) |
| 16. I would continue to attend TEAM Pain AD teleECHO clinics for pain assessment and management in dementia | 0 (0) | 0 (0) | 0 (0) | 6 (60) | 4 (40) |
| 17. I believe that TEAM Pain AD teleECHO clinics should be continued for pain assessment and management in other conditions | 0 (0) | 0 (0) | 1 (10) | 6 (60) | 3 (30) |
