# Supplementary material for: Evaluation of the impact of telementoring using ECHO© technology on healthcare professionals’ knowledge and self-efficacy in assessing and managing pain for people with advanced dementia nearing the end of life
Source: BMC Health Serv Res. 2018 Apr 2;18:228. doi: 10.1186/s12913-018-3032-y (PMC5879835; doi:10.1186/s12913-018-3032-y)
Supplement: Supplementary file 5 — Table S5. Post-ECHO evaluation responses: nurses. (DOCX 14 kb) [file 12913_2018_3032_MOESM5_ESM.docx]

**Additional file 5: Table S5. Post-ECHO evaluation: nurses**

| **Knowledge and self-efficacy evaluation statement** | **Number (%) of respondents who selected** | | | | |
| --- | --- | --- | --- | --- | --- |
|  | **Strongly**  **Disagree** | **Disagree** | **Neither Agree nor Disagree** | **Agree** | **Strongly**  **Agree** |
| 1. Participation in the teleECHO clinics has developed my **clinical knowledge** in **pain assessment** in advanced dementia | 0 (0) | 0 (0) | 1 (10) | 4 (40) | 5 (50) |
| 2. Participation in the teleECHO clinics has developed my **clinical skills** in **pain assessment** in advanced dementia | 0 (0) | 0 (0) | 2 (20) | 5 (50) | 3 (30) |
| 3. Participation in the teleECHO clinics has developed my **clinical knowledge** in **pain management** in advanced dementia | 0 (0) | 0 (0) | 2 (20) | 3 (30) | 5 (50) |
| 4. Participation in the teleECHO clinics has developed my **clinical skills** in **pain management** in advanced dementia | 0 (0) | 0 (0) | 2 (20) | 4 (40) | 4 (40) |

| 5. Did you present a patient case at a teleECHO clinic?  **IF YES**:  I am confident/comfortable presenting patient cases during teleECHO clinics  Presenting a patient case in the teleECHO clinics benefitted the patient in my care  **IF NO**:  I would be confident/ comfortable presenting patient cases during teleECHO clinics  I learned from providers who present their patient cases during teleECHO clinics | 0 (0)  0 (0)  0 (0)  0 (0) | 0 (0)  0 (0)  1 (12.5)  0 (0) | 0 (0)  0 (0)  1 (12.5)  0 (0) | 0 (0)  0 (0)  6 (75)  5 (62.5) | 2 (100)  2(100)  0 (0)  3 (37.5) |
| --- | --- | --- | --- | --- | --- |
| 6. I apply knowledge learned in teleECHO clinics to other patients in my care who have similar symptoms and diseases | 0 (0) | 0 (0) | 0 (0) | 6 (60) | 4 (40) |
| 7. I teach other clinical staff what I have learned in teleECHO clinics | 0 (0) | 0 (0) | 1 (10) | 7 (70) | 2 (20) |
| 8. Access to specialist expertise and consultation is an important area of need for me and my care staff team | 0 (0) | 0 (0) | 0 (0) | 3 (30) | 7 (70) |
| 9. Access to specialist expertise and health professionals from other specialties has been a benefit to my clinical practice | 0 (0) | 0 (0) | 0 (0) | 4 (40) | 6 (60) |
| 10. Access to expertise in pharmacology through the teleECHO clinics has benefitted my clinical knowledge and practice | 0 (0) | 0 (0) | 0 (0) | 4 (40) | 6 (60) |
| 11. Access to expertise in behaviour and mental health through the teleECHO clinics, has benefitted my clinical knowledge and practice | 0 (0) | 0 (0) | 0 (0) | 5 (50) | 5 (50) |
| 12. teleECHO clinics have improved the way that health professionals communicate with each other about pain in patients with advanced dementia nearing the end of life | 0 (0) | 0 (0) | 0 (0) | 4 (40) | 6 (60) |
| 13. Learning about complex chronic disease through participation in teleECHO clinics is an effective way to enhance clinical knowledge and expertise | 0 (0) | 0 (0) | 0 (0) | 4 (40) | 6 (60) |
| 14. Case-based learning as the focus for discussion is an impactful way of learning | 0 (0) | 0 (0) | 0 (0) | 4 (40) | 6 (60) |
| 15. Didactic sessions during teleECHO clinics were an effective way for me to develop my clinical knowledge and skills | 0 (0) | 0 (0) | 0 (0) | 5 (50) | 5 (50) |
| 16. I would continue to attend teleECHO clinics for pain assessment and management in dementia | 0 (0) | 0 (0) | 0 (0) | 5 (50) | 5 (50) |
| 17. I believe that teleECHO clinics should be continued for pain assessment and management in other conditions | 0 (0) | 0 (0) | 0 (0) | 3 (30) | 7 (70) |
| 18. I believe a separate teleECHO for nurses would be beneficial | 0 (0) | 3 (30) | 3 (30) | 3 (30) | 1 (10) |
